# Health risk assessment of methyl mercury from fish consumption in a sample of adult Qatari residents

**DOI:** 10.1007/s10661-023-11194-w

**Published:** 2023-04-27

**Authors:** Maetha M. Al-Sulaiti, Mohammad A. Al-Ghouti, Gouda A. Ramadan, Lama Soubra

**Affiliations:** 1 Environmental Science Program, Department of Biological and Environmental Sciences, College of Arts and Sciences, Qatar University, P.O. Box: 2713, Doha, State of Qatar; 2 Central Food Laboratories, Public Health Department, P.O. Box 42, Doha, Qatar; 3 Agricultural Research Center, Central Laboratory of Residue Analysis of Pesticides and Heavy Metals in Food, Ministry of Agriculture, Giza, 12311 Egypt

**Keywords:** Methyl mercury, Dietary exposure, Risk assessment, Vulnerable population, Fish consumption, Qatar

## Abstract

Fish constitutes an essential source of high-quality protein and is, at the same time, the source of exposure to many hazardous contaminants, namely mercury and methyl mercury (MeHg). This study aims at assessing the risk that MeHg poses to the health of adult Qatari residents through fish consumption. Data on fish consumption were collected using a self-administered online survey composed of three sections that collected information about the fish-eating patterns of the participants. The fish species that were reported to be consumed by ≥ 3% of the respondents were sampled and analyzed for their total mercury (T-Hg) content levels. MeHg concentrations were derived from T-Hg content levels using a scenario-based approach. Disaggregated fish consumption and contamination data were combined using the deterministic approach to estimate MeHg intakes. The average, 75th, and 95th percentiles of the MeHg intake estimates were determined and compared to the tolerable weekly intake (TWI) set by the European Food Safety Agency (EFSA) (1.3 μg·kg^−1^·w^−1^). All fish samples contained T-Hg at levels ˂ 0.3–0.5 µg/g with a mean value of 0.077 µg/g. The study population had an average fish consumption of 736.0 g/week. The average estimated weekly intakes of MeHg exceeded TWI for some fish consumers including females of childbearing age and those following a high-protein diet. Our study highlights the need to establish regulatory guidelines and dietary advice based on risk/benefit ratio.

## Introduction

Mercury (Hg), an ubiquitous environmental toxicant, is placed third on the 2019 Agency for Toxic Substances and Disease Registry (ATSDR) substance priority list because of its prevalence, toxicity, and high potential for human exposure (ATSDR, 2019). It is released into the environment from natural sources such as forest fires, volcanoes, and fossil fuels. Anthropogenic activities such as the incineration of municipal and medical wastes, industrial processes, and fossil fuel combustion can also release mercury into the environment (Al sulaiti et al., 2022). Once released into the atmosphere, mercury can be transported on a global scale, converted to other forms (Hg^+^, Hg^2+^), and/or returned to the soil and water by various depositional processes (Clarkson & Strain, 2020). In the aquatic system, part of the oxidized inorganic mercury (Hg^2+^) is methylated (Clarkson & Strain, 2020). The methylation process is believed to occur through a non-enzymatic reaction between Hg^2+^ and methyl-cobalamine produced by bacteria (Al-Sulaiti et al., 2022; Lin et al., 2021a, b; Grégoire et al., 2018). Formed methyl mercury (MeHg) can rapidly diffuse and bind to proteins in aquatic biota, leading to its bioaccumulation in fish and marine mammals (Al-Sulaiti et al., 2022; Lee & Fisher, 2017; Selin et al., 2009). MeHg also bio-magnifies within the food web, yielding higher MeHg concentration in large predatory fish species at the top of the aquatic food chain (Al-Sulaiti et al., 2022; Mehouel et al., 2019; Lee & Fisher, 2017; Selin, 2009). In addition, MeHg levels tend to be higher in older fish and fish of larger sizes within the same fish species (Magi et al., 2009). Although both inorganic and organic mercury may be found in fish, MeHg is the predominant form (Al-Sulaiti et al., 2022; Mehouel et al., 2019; Wei et al., 2018).

The contamination of fish by mercury and MeHg has been a major concern worldwide (Wei et al., 2018). On the one hand, fish is a main source of high-quality protein in the diet of humans around the world (FAO, 2022). In addition, fish is rich in omega 3, docosahexaenoic acid, linolenic acids, unsaturated fatty acids, minerals (selenium, iodine, magnesium, iron, copper, and vitamins (Jamioł-Milc et al., 2021; de Boer et al., 2020; Sofoulaki et al., 2018), which confer protective effects against non-communicable diseases (NCD) particularly cardiovascular diseases and rheumatoid arthritis and ensure normal neuronal development in children (Jamioł-Milc et al., 2021; Jubbin, 2016; Shapiro et al., 1996; Tedeschi et al., 2017; Hamazaki et al., 2020; Zeilmaker et al., 2013). On the other hand, fish consumption constitutes the main human exposure pathway to Hg and MeHg contaminants that have the potential to adversely affect human health (ATSDR, 2022). MeHg is the most toxic form (ATSDR, 2022; FAO/WHO, 2006), with a well-established neurotoxicity (ATSDR, 2022; FAO/WHO, 2006). Studies have shown that long-term exposure to low doses of MeHg was associated with developmental delays, learning disabilities, and possibly behavioral problems in fetuses, infants, and children, and with neurodegenerative disorders such as Parkinson’s and Alzheimer’s diseases in adults (ATSDR, 2022; FAO/WHO, 2006). Besides, evidence suggests that long-term exposure to MeHg may have negative effects on the immune and cardiovascular systems (ATSDR, 2022; FAO/WHO, 2006).

To achieve health benefits from fish consumption while protecting public health in general, and that of vulnerable groups in specific, international health agencies took many actions. First, a reference dose, which is determined to be a rate of exposure that a person can experience over a lifetime without appreciable risk of harm, was established for MeHg (FAO/WHO, 2006; EFSA, 2012). This reference dose was expressed in terms of tolerable weekly intake (TWI) and determined as 1.3 μg·kg^−1^·w^−1^ by European Food Safety Authority (EFSA) (EFSA, 2012).

This value was based on the neurodevelopmental effects as the critical effect in the developing fetus as the most sensitive sub-population (EFSA, 2012). Moreover, fish consumption advice and dietary guidelines were developed. Furthermore, maximum permissible limits of 0.5 µg/g and 1 µg/g for MeHg were determined in non-predatory and some predatory fish species respectively by JECFA and EFSA (FAO/WHO, 2006; the EU Commission, 2022), 0.3 µg/g by the US EPA (US EPA, 2017), and 0.3 µg/g in some fish species such as salmon by the EU commission (the EU Commission, 2022).

Many studies were conducted around the world to assess the health risks of MeHg from dietary exposure. The estimated risks varied by country depending on the amount and the type of fish consumed, as well as the fish contamination level (Ahmad et al., 2022; Barone et al., 2022; Domínguez-Morueco et al., 2022; Sarvan et al., 2021; Vasconcello et al., 2021; Petrova et al., 2020; Okati & Esmaili-sari, 2018; Wei et al., 2018; You et al., 2018; Zolfaghari, 2018; Laird et al., 2017; Malakootian et al., 2016; EFSA, 2012; Maycock & Benford, 2007).

In Qatar, fish constitutes a major food comodity (Canada, 2021). Moreover, there are limited data on the concentrations of mercury in fish and seafood available in Qatar (Elsayed et al., 2020; Al-Ansari, 2017). Previous studies focused on determining mercury contamination levels in limited marine fish species at different trophic levels that were collected from local sites. Moreover, according to our knowledge, no previous risk assessment for methyl mercury from fish consumption was conducted in Qatar, and very few were done in the GCC (Zolfaghari, 2018; Okati & Esmaili-sari, 2018; Laird et al., 2017; Malakootian et al., 2016). Since fish consumption patterns and contamination levels may differ across countries, it is difficult to infer conclusions on the MeHg risks from studies conducted elsewhere. Risk assessment studies, therefore, are needed in Qatar and the Middle East region not only to assess current risks from exposure to contaminants but also to draw legislative inferences (Saleh & Goktepe, 2019). Hence, this study was conducted to assess the risk that MeHg poses to the health of a sample of fish consumers and to determine whether the current permissible levels provide adequate protection for the health of fish consumers.

## Material and method

The study was conducted in Qatar between August 2021 and February 2022, after being approved by Qatar University institutional review board (IRB 1,807,049–1). The study population included adult Qatari residents aged ≥ 18 years old who are fish consumers.

### Fish consumption data

Data on fish consumption were collected using a self-administered online survey that was specifically designed to serve the purpose of this study. The survey was initially developed by an expert in the field of risk assessment in the English language. The survey was reviewed by a committee composed of two experts in the field of nutrition and adjusted based on their comments. The survey was then translated to the Arabic language and back-translated to the English language. The discrepancies between the two versions were reviewed and adjusted accordingly. Moreover, the final forms of the survey (English and Arabic) were pilot tested on a sample composed of twenty adults to check for clarity and the time required to be completed. The surveys collected from the pilot testing were not included in the final sample.

The survey was composed of three parts. The first part collected information about the participant’s age, sex, body weight, body height, pregnancy status (for female participants), and area of residency. The second part asked about the food diet of the participant, more specifically whether the participant was following a specific type of diet (low-carb, ketogenic, high protein, etc.…); the fish species commonly consumed; the main place(s) for fish purchasing (with their names), i.e., supermarkets, fish markets, fishermen; and the typical frequency of eating fish in a week time frame.

The third part consisted of a fish consumption questionnaire. The questionnaire was designed to collect information about the frequency and the amounts of fish species consumed during a week time frame, based on a reference period of the previous year. The fish species that were included in the questionnaire were those reported to be the most commonly consumed in Qatar (Sana et al., 2020). These were HM, SF, CH, SH, TU, SM, SB, ANCH, and SAR. To avoid confusion between fish species, each fish species was presented using its common name and its picture. Furthermore, participants had the option to report the consumption of other species than those listed by selecting the “other species” option. The fish consumption frequency responses were never, one time per month, two times per month, three times per month, one time per week, two times per week, three times per week, four times per week, five times per week, six times per week, once per day, twice per day, and three times per day. Here also, the participants had the opportunity to report an eating frequency outside those listed. The medium size portion was used as the standard portion and consumed amounts were derived from this portion as 1/3, half, one, two, three, and four times the portion size. Participants were also able to report amounts outside the provided amount range. Besides, the questionnaire included a picture of a standard portion (with its weight, 150 g (average fillet weight for fresh fish) and 120 g (drained weight) for canned TU, and 150 g (drain weight) for canned SM) for each of the fish species to facilitate on the participants the estimation of their consumed amounts. In addition, the participants were asked to report for each fish species the ways it was purchased, i.e., fresh, frozen, canned, whole, fillet, cuts, etc.; the commonly purchased brand names for frozen and canned fish; and the origins of fresh fish, i.e., locally caught or imported.

The survey was prepared using Google Forms^®^. The survey was completed online by the participants without any support or intervention. An invitation to participate in the survey, together with its link, was sent to the public through e-mails and social media platforms. In the invitation, the purpose of the survey as well as the eligibility criteria to participate in the survey was clearly stated. Besides, the participants had to provide consent before starting taking the survey. The invitation was sent in many rounds until achieving the required sample size. The sample size was calculated based on around 2,400,000 adult population living in Qatar, with a 95% confidence interval, and a 5% error margin yielding 385 respondents. The sample was further increased to 660 to account for errors in filling the questionnaire and to allow analysis of subgroups. Collected surveys were reviewed for their completeness and correctness. In case of an incomplete survey or errors in filling in the survey, such as if there was a discrepancy between the information provided in the second section and that provided in the third (frequency of fish-eating for example), the survey was discarded. Data from complete surveys were entered into an Excel sheet for further analysis. All entered data were double-checked for accuracy by random check of entered data and survey responses.

Data were analyzed to determine the most commonly consumed fish species, the main forms in which they were bought, and the main purchase places. In addition, for each participant, the amount of each of the fish species consumed and the total amount of fish consumed (expressed in gram/week (g/w)) were calculated. The mean, 75th, and 95th percentile of the weekly fish consumption distribution were then derived for the group of participants. Besides, to take into account the variability in fish consumption among different ethnicities and sex the respondents were divided into two cohorts: Qatari and non-Qatari nationals. The two cohorts were then divided into female, male, and high-protein diet groups. Moreover, since the females of the childbearing age are the most vulnerable group (EFSA, 2012), the female cohort was further divided into two subgroups based on their age: childbearing age and non-childbearing age. The childbearing age was considered between 18 and 40 years based on the WHO categorization (15–40 years) with adaptation to the specific cultural context of the region (WHO, 2021). The mean, 75th, and 95th percentile of the weekly fish consumption distribution were also calculated for each of the cohorts and subgroups.

### Sample collection and preparation

Sixty-five composite fish samples were prepared and analyzed for their content in total mercury (T-Hg). Table 1 presents a description of the collected fish samples. In brief, in order to ensure the representativeness of the collected samples, many points were addressed in the design of the sampling plan. First, the fish species that were reported to be consumed by ≥ 3% of the respondents were included in the sampling scheme. The fish species were coded as the following: HM, SF, SH, CH, TU, SB, and SM. The samples were collected in the most frequently reported purchased forms for each of the fish species (i.e., fresh, frozen, whole, fillet, etc.). Besides, they were bought from the locations that were determined from the survey responses as the most common fish purchase places. For each fish species bought from a specific place, three samples (small, medium, and large size) were collected. The fish samples were randomly collected on different days in two different periods during the study timeline based on their availability to cover seasonal variability. All fish samples were transported in dry-sealed plastic zipper bags and placed in an ice pack to the laboratory. Each fish sample was appropriately labeled at the time of purchase. In the laboratory, edible muscle and skin were collected using a steel knife and plastic cutting board to avoid contamination during sample preparation. Multiple samples obtained for the same fish species from the commonly purchased locations across the country in the specific season are composited. Composite samples were homogenized, weighed, and dried on an aluminum tray in an oven at 80 °C (Kendro Laboratory Products Heraeus UT 20 oven). Dried composite samples were weighed and ground using a stainless steel food processor made of stainless-steel bowls and blades (Panasonic, Model No. MX-GX1061, Panasonic Taiwan Co. Ltd., China). To avoid cross-contamination or carry-over between samples, the food processor was cleaned thoroughly before and after grinding each sample. The composite samples were labeled and stored in plastic zipper bags at −20 °C until the analysis time. The moisture content range for each of the fish species was determined using the following equation (Eq. 1):

**Table 1:** Collected fish samples

**Type**	**Common name—scientific name**	**Trophic level**	**Local or imported**	**Wild or farmed**	**Whole or fillet**	**Average fish or fillet plate weight (g) ± SD**	**Number of samples**
Fresh	HM—*Epinephelus coioides*	Carnivores	Local	Wild	Whole	1405 (123)	3
							3
			Imported	Unknown	Fillet	374 (35)	3
	SF—*Siganus rivulatus*	Herbivores	Local	Wild	Whole	217 (40)	3
							3
	CH*—Scomberomorus commerson*	Carnivores	Local	Wild	Whole	1450 (210)	3
							3
	SH—*Lethrinus nebulosus*	Carnivores	Local	Wild	Whole	775 (159)	3
							3
	TU (local: *Euthynnus affinis)*	Carnivores	Local	Wild	Whole	1443 (354)	3
			Imported	Unknown	Fillet	165 (34)	3
	SM—unknown	Omnivores	Imported	Farmed	Fillet	328 (26)	3
							3
	SB—*Dicentrarchus labrax*	Carnivores	Imported	Farmed	Whole	508 (38)	3
							3
Frozen	SM—*Salmo salar*	Omnivores	Imported	Unknown	Fillet	498 (43)	1
Canned	TU—different species	Carnivores	Imported	Unknown	Can	-	16
	SM—unknown	Omnivores	Imported	2 wild and 1 unknown	Can	-	3
						Total	65

1 $$\mathrm{Moisture}\;\mathrm{content}=100-\left[\left(\mathrm{dry}\;\mathrm{weight}/\mathrm{wet}\;\mathrm{weight}\right)\times100\right]$$

### Sample analysis

The analysis of composite fish samples for the determination of their T-Hg content was conducted in the central food laboratory unit of the ministry of public health. The analysis procedure was performed according to the food laboratory standard operating procedures (SOPs) QMS code CTS-18 and using 1% nitric acid (HNO_3_) as blank (supplied from Fluka Analytical).

All reagents used in this study were of analytical grade. All plastic- and glassware were soaked in nitric oxide 10% for 24 h and thoroughly rinsed with deionized water (8 MΩ-cm (Elix/Milli-Q, Millipore, USA). Deionized water was used in the dilution of samples, preparation of reagent blanks, and calibration standards. Nitric acid solution (HNO_3_) 1% was prepared from 65 to 70% HNO_3_ from Fluka Analytical. Mercury stock solution was prepared from 1000 ppm Hg and gold (Au) 50 ppm from Perkin Elmer. Au was used for the stabilization of the calibration curve standards. A hydrochloric acid solution (Scharlau) (1.5 M) and ethanol (Oxford lab) were used for washing. Standards, intermediate, and spiking solutions were prepared using disposable polypropylene tubes (15 mL and 50 mL), and an automatic micropipette 20–200 µL and 100–1000 µL.

Briefly, samples were subjected to acid digestion using an ultra-WAVE microwave digestion system. This was done by accurately weighing about 0.2500 g of the dried sample to the nearest 0.1 mg using an analytical sensitive balance (Mettler Toledo) inside a microwave (MW) Teflon digestion vessel and then adding 4.0 mL of concentrated HNO_3_. The samples were digested for 30 min under 110 bar at 60 °C to 220 °C and then cooled to room temperature for 30 min. After digestion, the sample solutions were transferred into 50-mL clean graduated Falcon tubes (Fisher Scientific, USA). The Teflon vessels were washed several times with deionized water and the washings were added to the graduated tubes. The solutions were diluted with deionized water to a total volume of 25 mL.

Total Hg levels in samples were determined using an in-house validated inductively coupled plasma mass spectrometry (ICP-MS) method (Perkin Elmer NexION 350, equipped with an elemental scientific autosampler, and Syngistix software). The standard working solutions (concentrations series 0, 0.5, 10, 50, 100, 500 ng/mL) were prepared according to the Ministry of Public Health standard Operating procedures (SOPs) Quality management system (QMS) code CTS-18 method using 1000 ppm Hg and gold (Au) 50 ppm for stabilization (supplied from Perkin Elmer). The accuracy of the method was validated by analyzing certified reference materials (CRM): CRM 7279—canned crab meat and CRM 7271—canned fish. All CRMs were stored under the same conditions, prepared, and digested with the same protocol as the analyzed fish samples. The described method validation parameters were as follows: the limit of detection (LOD) was 1 µg/kg; the limit of quantitation (LOQ) was 10 µg/kg; the recovery rates were between 95.07 and 115.3%; the relative standard deviation (RSD) was 3.32%; and the linearity range was 10 µg/L and 500 µg/L with a correlation coefficient ≥ 0.99. The quality control was done using a calibration curve, spiked samples, and continuing calibration verification (CCV). Two blank samples were analyzed together with each sample batch. T-Hg concentrations in blanks were below the detection limits in all the analyses. Blanks and working standard solutions were analyzed in a similar way to the digested sample solution, and calibration curves were constructed. Analyses were duplicated to check the reproducibility of the results. Relative standard deviations among replicates were always less than 10%. Recovery tests were done in selected samples by spiking analyzed samples with aliquots of standards and then carrying out digestion. The recovery ranged from 104 to 114%. The CCV was prepared by injecting a mid-range calibration standard to ensure the validity of the initial calibration of the instrument. Quality control tests were run every 15 samples. The T-Hg of the fish (wet weight) was derived from the measured T-Hg of the dried samples using the following formula (Eq. 2):

2 $$\mathrm T-\mathrm{Hg}\;\mathrm{concentration}\;\left(\mathrm{wet}\;\mathrm{weight}\right)\;=\;\left[\frac{\left(100\;-\;\mathrm{moisture}\;\mathrm{content}\right)}{100}\right]\;\times\;\mathrm T-\mathrm{HG}\;\mathrm{concentration}\;\left(\mathrm{dry}\;\mathrm{weight}\right)$$

### Exposure assessment

The exposure to MeHg was determined based on the disaggregated fish consumption data and the contamination data determined by chemical analysis of fish samples (WHO, 2008; US EPA, 2001; FAO/WHO, 1997). When the fish sample contained T-Hg below the quantification limit, it was assumed that T-Hg content is equal to LOQ/2. The disaggregated method considers each fish species as a food item with a well-defined contamination level, whereas in the aggregated method, all fish species are grouped in one group and an average contamination value is attributed to the whole group. The disaggregated method was chosen to avoid overestimation of exposure resulting from the use of aggregated data (FAO/WHO, 1997).

Moreover, the contamination levels of fish with MeHg were derived from their content in T-Hg determined by analysis using a scenario-based approach. In the first scenario, which is the worst-case scenario, it was assumed that all T-Hg is MeHg. In the second scenario, the percentages reported in the literature for each fish species were used to convert from T-Hg to MeHg (Table 2).

**Table 2:** Percentage of MeHg from T-Hg in the seven species

Species	MeHg %	References
HM	97.3	(Freije & Awadh, 2009)
SH	94.1	(Freije & Awadh,, 2009)
CH	97.6	(Freije & Awadh,, 2009)
SF	94.5	(Burger et al., 2014)
TU (mackerel)	93.0	(Ahmad et al., 2022)
TU (yellowfin)	96.3	(Nicklisch et al., 2017)
Canned TU	90.5	(de Paiva et al., 2017; Dezfouli et al., 2018)
SM (fresh and canned)	80.5	(Afonso et al., 2015; Sarvan et al., 2021)
SB	81.1	(Maulvault et al., 2016)

A third scenario was used to check whether the current maximum permissible levels set by the EU guidelines are protective for the health of fish consumers in Qatar. In that scenario, it was assumed that all fish species and selected predatory fish species, such as TU, are contaminated with mercury at their maximum allowed levels, i.e., 0.3 µg/g, 0.5 µg/g, and 1 µg/g, respectively.

In line with other studies, the exposure to MeHg was determined using the deterministic approach. The exposure was calculated for each participant, in each scenario, by summing the consumption data of the individual fish species multiplied by their average contamination levels data obtained from the analysis and dividing them by the participant’s actual body weight using the following Eq. (3):

3 $$\mathrm{MeHg}\;EWI(p)(\frac{\mathrm\mu\mathrm g/\mathrm{week}}{\mathrm{kg\;b}.\mathrm w.})=\sum\nolimits_i^n(\mathrm{CONS}^i\times\mathrm{CONT}^i)/{\mathrm B.\mathrm W.}^p$$

where estimated weekly intakes (EWI) is the estimated weekly intake μg·kg^−1^·w^−1^ for the participant *p*, CONS(*i*) is the consumption (g/week) for a specific fish species, CONT(*i*) is the average MeHg contamination level (µg/g) of that fish species (*i*), *n* is the number of consumed fish species by the participant *p*, and B.W. (*p*) is the body weight (kg) of the participant *p*.

The average, 75th (P75), and 95th (P95) percentiles of MeHg intake estimates were then derived for the different cohorts in the different scenarios. In addition, the percentage of contribution of each fish species to the MeHg estimated intake was determined using the following Eq. (4):

4 $$\%\;C\;(i)=\frac{EWI\;(i)}{EWI\;(T)}\times100$$

where %C (*i*) is the percentage of contribution of the fish species (*i*) to the exposure to MeHg, EWI (*i*) is the estimated weekly intake from the fish species (*i*) (μg.kg^−1^.w^−1^), and EWI (*T*) is the estimated weekly intake from all fish species (μg.kg^−1^.w^−1^).

### Risk characterization

The risk was characterized for the different cohorts considering the two exposure scenarios. This was done by calculating the hazard quotient (HQ) using the following Eq. (5):

5 $$HQ=\frac{EWI }{TWI}$$

where HQ is the hazard quotient, EWI is the estimated weekly intake (μg.kg^−1^.w^−1^), and TWI is the tolerable weekly intake (reference dose) proposed by the EFSA (1.3 μg.kg^−1^.w^−1^).

The HQ was compared to 1 to estimate the risk. When the HQ is less than 1, the risk of MeHg toxicity from fish consumption is considered negligible, whereas when the HQ is equal to or greater than 1, the risk of MeHg toxicity due to fish consumption is considerable. Moreover, the higher the HQ, the greater the risk of toxicity. The percentages of the EWI to the TWI were then derived.

In this study, the risk was also quantified by estimating the likelihood of a given population exceeding the TWI under the scenarios applied (Eq. 6).

6 $$P(EWI\geq R\;f\;D)=\frac{\#\;(EWI\geq RfD)}N$$

The percentage risk of exceeding the toxicological reference value (TRV) was obtained by calculating the ratio between the number of consumers exceeding it and the total number of subjects studied under each of the applied scenarios. In other words, this percentage corresponded simply to the number of individuals exposed to an identified hazard whose mean theoretical exposure also exceeded the toxicological threshold corresponding to this hazard.

### Risk management

#### Estimation of safe fish consumption rates

To propose sound recommendations for MeHg risk management, the safe fish consumption rates (g/week) that would be consumed without posing a risk to the health of fish consumers were determined for the fish species that are the main contributors to MeHg exposure. The calculation was based on the average exposure estimates and was done using the contamination data of the most contaminated sample of the fish species. It was done for “all participants” and “high-protein diet” groups, and the “females of childbearing age” subgroup. It was calculated using the following equation (Eq. 7) (US EPA, 2000):

7 $$CRs_i=(TRV\;X\;B.W)/CONT$$

where $CR{s}_{i}$ is the safe consumption rate of the fish species (g/week), TRV is the tolerable weekly intake (reference dose) proposed by the EFSA (1.3 μg.kg^−1^.w^−1^), BW is the consumer average body weight (76.8 kg (all participants), 67.3 kg (participants on high-protein diet), and 68.9 (females of the childbearing age)), and CONT (is the maximum recorded contamination level of fish samples for the fish species (μg/g)).

#### Estimation of MeHg threshold limits in fish based on current fish consumption data

The MeHg threshold limit in fish was also determined. It was done based on average fish consumption estimates of the “females of childbearing age” subgroup since they are the most vulnerable group of the population. It was calculated using the following equation (Eq. 8) (US EPA, 2000):

8 $$TV=(TRV\;X\;B.W)/CONS$$

where TV is the threshold value (μg/g) of MeHg in fish. TRV is the Toxicological reference value (tolerable weekly intake) proposed by the EFSA (1.3 μg.kg^−1^.w^−1^), BW is the females of childbearing age average body weight (68.9 kg), and CONS (is the average fish consumption of the females of the childbearing age (g/w)).

### Statistical analysis

All data were entered into an excel sheet and analyzed using the Minitab^®^ 20. All entered data were doubled-checked for accuracy by random comparison between the collected data and the data entered. Descriptive statistics were used to assess data normality using the one-sample Kolmogorov–Smirnov test. Means and standard deviations (SD) were calculated for continuous variables and compared using the ANOVA test. A *p*-value of ≤ 0.05 was used to indicate statistical significance.

## Results and Discussion

### Participants

Six hundred eighty-nine adults participated in the survey. Eighty-nine surveys were discarded because of obvious errors in responses. Table 3 presents the main characteristics of the participants. Fifty-six percent (336) were Qatari, 64% (426) were females, and 19.6% (118) were following a high-protein diet. Moreover, the participants had an average age of 35.8 ± 10.9 years and an average Body mass index (BMI) of 28 ± 6.5 kg/m^2^. There was no statistically significant difference between the age of male and female participants. Both sexes had a BMI that falls within the overweight category. Among females of childbearing age, 3.2% (19) were pregnant and 4.3% (18) were breastfeeding.

**Table 3:** Participants’ characteristics

**Characteristics**	**All participants**
**Total *N* (%)**	600 (100)
**Average age mean ± SD**	35.8 ± 10.9
**Average body weight (kg)**	
** Mean ± SD**	76.8 ± 20
** Median**	73.5
** Range**	149
**Average height (cm)**	
** Mean ± SD**	164.9 ± 10
** Median**	163
** Range**	124
**Average BMI**	
** Mean ± SD**	28 ± 6.5
** Median**	27.3
** Range**	54.8
**Nationality *N* (%)**	
** Qatari**	336 (56)
** Non-Qatari**	264 (44)
**Sex *N* (%)**	
** Females**	426 (64)
**Childbearing age**	226 (37.6)
**Pregnant**	19 (3.2)
**Breastfeeding**	18 (3)
**On high-protein diet**	73 (12.2)
**Males**	174 (26)
**Males on high-protein diet**	45 (7.5)
**Total *N* (%)**	600 (100)

### Fish consumption

**Fig. 1:**
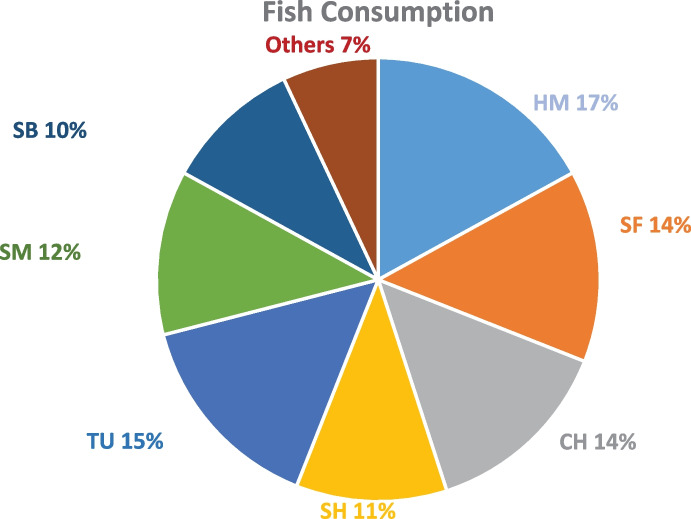
Consumed fish species

The main fish species that were reported to be consumed by the participants are shown in Fig. 1. The most consumed species were HM 17% (of total consumed fish (2752)), followed by TU 15%, SF 14%, CH 14%, SM 12%, SH 11%, and SB 10%. Fish were mostly bought as fresh for all species (99.5%) except for TU, which was mostly bought as canned (93%). The only fish species that was reported to be bought frozen was SM. The findings of this study agreed with a previous study where it was reported that the Qatari residents preferred fresh fish over frozen ones (Sana et al., 2020).

The average, p75, and p95 of the fish consumption rates for the “all participants,” “Qatari” and “non-Qatari” cohorts, “male” and “female” groups, and female subgroups are presented in Table 4. The average, median, and ranges of fish consumption rate of all participants were 735.96, 657, and (340-1651) g/w respectively. The average fish consumption rates were significantly different between the “Qatari” and “non-Qatari” cohorts (858.36 ± 605 versus 622.31 ± 542, *p*-value: 0.001), but did not differ significantly between females and males (756.70 ± 605 versus 734.10 ± 582, *p*-value: 0.154). Besides, in the female group, females of the childbearing age subgroup had significantly higher average fish consumption than that of the non-childbearing age (880.18 ± 765 versus 708.29 ± 582, *p*-value: 0.154). The p75 of fish consumption for all participants, females, males, participants on a high-protein diet, and females of childbearing age were 962.75 g/w, 994.12 g/w, 890.54 g/w, 1224.12 g/w, and 1101.54 g/w respectively. The p95 of fish consumption for all participants, females, males, participants on a high-protein diet, and females within the childbearing age were 1161.86 g/w, 1285.86 g/w, 1036.92 g/w, 1502.56 g/w, and 1358.94 g/w respectively. Females of childbearing age and participants on a high-protein diet had significantly higher average, p75, and p95 fish consumption rates than all other groups (*p*-value: 0.001).

**Table 4:** Average, p75, p 95 of Fish consumption rates per week in g/week/person

**Groups**	**Fish consumption rates (g/week ± SD)**		
	**Average**	**p75**	**p95**
**All participants**	735.96 ± 634	962.75 ± 673	1161.86 ± 582
**Qatari cohort**	858.36 ± 605	1090.12 ± 675	1290.12 ± 675
**Non-Qatari cohort**	622.31 ± 542	810.23 ± 573	1032.23 ± 473
**High-protein diet group**	880.18 ± 765	1224.06 ± 565	1502.56 ± 865
**Male group**	734.10 ± 542	890.54 ± 503	1036.92 ± 554
**Female group**	756.70 ± 605	994.12 ± 614	1285.86 ± 675
**Female of child-bearing age subgroup**	822.56 ± 865	1101.99 ± 607	1358.94 ± 810
**Female of non-childbearing age subgroup**	708.29 ± 582	898.54 ± 630	1213.30 ± 456

The average fish consumption yields an average fish consumption of 38.22 kg per capita/year, which is around two times higher than the range of 22.3 kg per capita/year reported by the Food and Agriculture Organization (FAO) (FAO, 2022). This difference may be explained by the difference in the method used to estimate fish consumption. In FAO estimates the consumption is based on balance sheets and is expressed per capita, whereas, in this study, estimates were done for fish consumer-only using their dietary records. On a theoretical level, a consumer-only fish consumption estimate is the rate for the population that consumes fish, excluding those who do not, while the per-capita estimate is the fish consumption rate for the entire population, both consumers and non-consumers. The use of per-capita estimates would yield a more flattened consumption rate (FAO/WHO, 2009). Besides, the high consumption rates observed in the females of childbearing age can significantly contribute, in the case of pregnancy, to the in utero exposure of the fetus to the contaminants contained in the fish. It can therefore impose an increased risk of neurotoxicity on the embryo and fetus if fish contamination by mercury was above the guidelines levels (EFSA, 2012).

The average (± SD) fish consumption rates (g/week/person) of the selected fish species for all participants and the Qatari and non-Qatari cohorts are shown in Table 5. HM was the most consumed fish species in the “all participants” group, with a weekly consumption mean of 179.0 ± 187 g/w and a percentage of consumers of 83%, whereas SF was the most consumed fish species in the Qatari cohort (mean 232.9 ± 205 g/w, percentage of consumers 87.5%), and SM in the non-Qatari cohort (mean 154.2 ± 181 g/w, percentage of consumers 78.8%). It is well known that fish choice and consumption are highly influenced by the culture, price, and availability in the market (Ikem & Egiebor, 2005). Therefore, fish consumption patterns would differ between populations, ethnicities, and countries. Consequently, the amounts and consumed fish species are expected to be variable between the different study groups. Fish consumption rates were similar to those reported by other countries in the GCC and outside the GCC, where fish is the main food commodity (Ahmad et al., 2022; Lin et al., 2021a, b; Laird et al., 2017, Ahmad et al., 2016; Burger et al., 2014), while they were higher than rates reported in the European countries where fish is not the main food commodity (Barone et al., 2022; Lofstedt et al., 2021; Farmery et al., 2018; Zolfaghari, 2018, Adeli, 2013). Besides, despite comparable fish consumption rates, the types of consumed fish species were different from those reported in these studies. Yet, similar to this study, HM was reported to be the most consumed fish species in the studies conducted in the GCC countries (Laird et al., 2017; Ahmad et al., 2016; Burger et al., 2014).

**Table 5:** Fish consumption per week (CONS) in g/week (mean ± SD) and the percentage of consumers (C%) of the selected fish species for “all participants” and “Qatari” and “non-Qatari” cohorts

**All participants** **(** *n* ** = 600)**			**Qatari cohort** **(** *n* ** = 336)**		**Non-Qatari cohort** **(** *n* ** = 264)**	
**Fish species**	**Fish CONS**	%C*	**Fish CONS (g/w)**	%C*	**Fish CONS**	%C*
**HM**	179.0 ± 187	83.0	198.4 ± 190	86.3	154.2 ± 181	78.8
**SF**	178.7 ± 213	69.3	232.9 ± 205	87.5	109.8 ± 174	46.2
**CH**	145.7 ± 209	71.3	200.8 ± 204	82.4	95.5 ± 178	57.2
**SH**	127.0 ± 277	55.2	127.9 ± 341	52.1	125.9 ± 169	59.1
**TU**	79.45 ± 121	72.3	142.8 ± 271	68.5	90.89 ± 141	76.1
**TU (canned)**	92.4 ± 154	90.2	94.67 ± 161	90.7	178.1 ± 186	89.7
**SM**	173.0 ± 288	56.8	165.1 ± 288	54.2	183.0 ± 196	60.2
**SM (canned)**	13.5 ± 21.3	5.85	12 ± 16.7	6.8	15.3 ± 12.3	4.9
**SB**	111.9 ± 182	51.3	118.1 ± 187	54.2	104.1 ± 190	47.7

*%C: percentage of fish species consumers

### Fish contamination levels

**Fig. 2:**
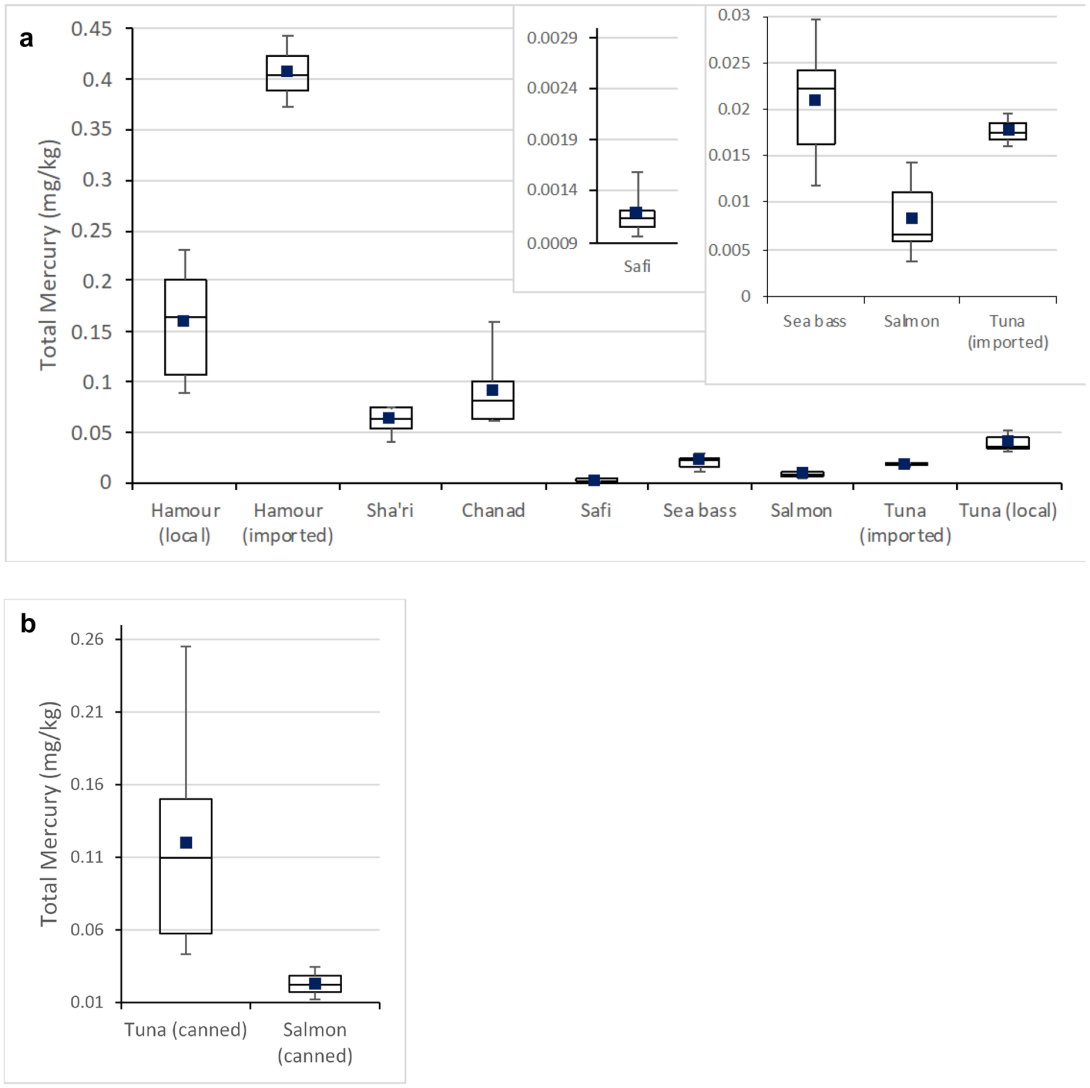
Box-and-whisker plot of HgT concentrations (mg/kg) (wet weight) for seven fish species. **a** The concentration of HgT in the fresh samples; and **b** the concentration of HgT in the canned samples. The two interquartile boxes represent the Q1 and Q3 separated by the median, the blue squares near the middle of the box are the average values, the whisker represents the error bars

Mean T-Hg concentrations (SD) and their ranges in the analyzed fish samples are presented in Table 6 and Fig. 2. T-Hg was detected in all analyzed samples. All analyzed fish samples contained T-Hg within the quantification limits except in SF. The mean (SD) T-Hg concentration of all fish species was 0.366 (0.58) µg/g (dry weight) and 0.083 (0. 09) µg/g (wet weight). The mean T-Hg concentrations (SD) ranged from 0.005 (0.001) µg/g in SF to 2.109 (0.18) µg/g in imported HM, corresponding to 0.001 µg/g wet weight and 0.406 (0.04) µg/g in SF and imported HM respectively. The means and ranges of T-Hg concentrations in all collected fish samples were within the EU guidelines ranges (0.3–0.5 µg/g wet weight; EU Commission, 2021). Besides, significant variations in the T-Hg concentration levels existed in the different fish species, *p*-value ≤ 0.05). Imported HM had the highest average T-Hg concentration with a value of 0.406 ± 0.04 µg/g, followed by local HM (0.158 ± 0.06 µg/g), canned TU (0.091 ± 0.07 µg/g), CH (0.091 ± 0.04 µg/g), SH (0.063 ± 0.01 µg/g), local TU (0.040 ± 0.01), canned SM (0.023 ± 0.01), SB (0.021 ± 0.007), SM (0.008 ± 0.004), and SF (0.0012 ± 0.0002 µg/g). Carnivorous fish were found to contain significantly higher levels of T-Hg than omnivorous and herbivorous ones (*p*-value ≤ 0.05). Besides, among carnivorous fish, SB had the lowest mean T-Hg concentration (*p*-value ≤ 0.05). Moreover, imported HM had a significantly higher mean T-Hg concentration than the local one (*p*-value ≤ 0.05), whereas local TU had a significantly higher concentration than the imported one (*p*-value ≤ 0.05). Finally, canned fish had significantly higher T-Hg mean concentrations than the fresh fish of the same species. Variable T-Hg concentrations in fish were reported in the literature. These variations may be related to the sampled fish (species, age, and size), sampling sites, and season (Da Silva et al., 2020; Elsayed et al., 2020, Wei et al., 2018). Nevertheless, previous studies conducted in the Gulf region reported similar results (Elsayed et al., 2020; Al-Ansari, 2017; Laird et al., 2017; Ahmad et al., 2016; Burger et al., 2014). All of the commercially available fish species in Qatar are considered to have T-Hg levels within acceptable levels set by EU. Besides, the finding that T-Hg concentrations decreased within the following order: carnivores˃ omnivores˃ herbivores, agrees with the fact that Hg concentrations vary according to the trophic level, with higher levels found in species found at higher trophic levels (Elsayed, 2020). SF is a non-predatory herbivore that feeds on algae and seagrasses which make this species at a lower trophic level. Those characteristics explain the significantly low T-Hg concentration (Soykan et al., 2020). T-Hg concentration in fresh SM, an omnivorous species, ranged between 0.004 and 0.014 mg/kg. In general, SM is considered to contain low Hg levels and is stated among the best choices category by the EPA and FDA (USDA, 2021). However, this needs to be balanced against higher levels of lipophilic contaminants as compared to lean fish filet (Quang et al., 2021). Moreover, HM had the highest T-Hg concentrations. This finding is in agreement with previous studies where T-Hg concentrations in HM were found to be the highest and exceeded the guidelines levels in some samples (Elsayed et al., 2020; Al-Ansari et al., 2017; Laird et al., 2017; Ahmad et al., 2016; Burger et al., 2014). HM is a carnivorous predatory fish species that is placed at the top level of the trophic chain (Wei et al., 2018). Mercury is known to bioaccumulate and biomagnify in fish, and thus relatively high concentrations can be attained in top predators, such as HM. Although all fish samples contained T-Hg below the guidelines limit levels (0.5–1 µg/g), three samples of HM exceeded the guidelines limit level of 0.3 µg/g set to protect vulnerable people, including pregnant women, children under 15 years old, and those who consume two or more servings of fish per week (US EPA, 2017). Therefore, to achieve the health benefits associated with fish consumption and to protect against neurodevelopmental toxicity of methylmercury, the consumption of imported HM species with a high content of mercury should be limited. In addition, the variability in T-Hg concentrations within the same species from different regions can be associated with different pollution levels of the fishing sites (Wei et al., 2018). Besides, results of this study demonstrated that canned fish had significantly higher T-Hg concentrations than non-canned ones. Canning process was reported to increase Hg concentration by 23%, due to decrease in the moisture ratio (Afonso et al., 2015; Helberg & Morrissey, 2007). Moreover, these differences may be attributed to the difference in fish species and fishing areas. Fish caught in the Atlantic Ocean presented higher mercury content than the ones from closed seas (Pawlaczyk et al., 2020). In addition, T-Hg concentrations might also be affected by the size/age of the fish species (Wei et al., 2018).

**Table 6:** Total Hg (THg) concentration (mean ± SD) and ranges of the collected fish samples (mg/kg) WW

**Fish species**	**Dry weight** **Mean ± SD**	**Wet weight** **Mean ± SD**	**Range** **(Wet weight)**	**Moisture content range (%)**
HM (local)	0.670 ± 0.25	0.161 ± 0.06	0.089–0.230	75–77
HM (imported)	2.109 ± 0.18	0.422 ± 0.04	0.372–0.443	80–81
HM (all)	1.39 ± 0.49	0.333 ± 0.07	0.089–0.443	75–77
SF	0.005 ± 0.001	0.001^a^ ± 0.0002	0.0009–0.002	76–78
CH	0.332 ± 0.14	0.091 ± 0.04	0.06–0.161	72–73
SH	0.278 ± 0.06	0.064 ± 0.01	0.042–0.076	74–81
TU (local)	0.132 ± 0.05	0.040 ± 0.01	0.032–0.052	67–71
TU (imported)	0.085 ± 0.004	0.018 ± 0.002	0.016–0.020	78–80
TU (all)	0.11 ± 0.234	0.029 ± 0.009	0.016–0.052	67–80
TU (canned)	0.273 ± 0.2	0.092 ± 0.07	0.015–0.255	59–75
SM	0.024 ± 0.01	0.008 ± 0.004	0.004–0.014	62–69
SM (canned)	0.070 ± 0.04	0.023 ± 0.01	0.012–0.035	64–69
SB	0.055 ± 0.02	0.022 ± 0.007	0.012–0.030	53–65

^a^SF had THg below the limit of quantification (0.01 mg/kg). Therefore, the value was replaced by LOQ/2

### Methyl mercury concentrations

The average concentration of MeHg from the three scenarios is presented in Table 7. The average MeHg concentrations ranged from 0.001 to 0.406 µg/g ww in scenario 1 and from 0.000945 to 0.395 µg/g ww in scenario 2. In both scenarios, all samples had MeHg concentrations below the guidelines limit levels (0.3–1 µg/g). Hence, the most consumed fish species in Qatar carry a low risk of contamination with MeHg. Nevertheless, this finding should be interpreted with caution since both fish contamination patterns and contamination levels can have divergent results based on changes in eating preferences over time, the fish age/size, fishing sites, and season (Al-Sulaiti et al., 2022).

**Table 7:** MeHg average concentrations in fish species in (µg/g WW) for the applied scenarios

**Fish species**	**Scenario 1**^**a**^	**Scenario 2**^**b**^	**Scenario 3**^**c**^
HM	0.333	0.324	0.5
SF	0.001	0.000945	0.5
CH	0.091	0.089	0.5
SH	0.064	0.06	0.5
TU	0.029	0.027	1
TU (canned)	0.092	0.083	1
SM	0.008	0.007	0.5
SM (canned)	0.023	0.019	0.5
SB	0.021	0.018	0.5

^a^Scenario 1: 100% of T-Hg were assumed to be MeHg

^b^Scenario 2: T-Hg were assumed to be MeHg using percentages reported in the literature

^c^Scenario 3: Fish were assumed to contain MeHg at the maximum permissible levels (0.5 ppm for all fish species, 1 ppm for Tuna) according to EU guidelines

### Exposure assessment

The contributions of each fish species to the total MeHg intakes for the “all participants,” “Qatari” and “non-Qatari” cohorts and female of childbearing age subgroup are presented in Tables 8 and 9. As shown in the tables, the main contributors to MeHg intakes are HM and canned TU with a contribution varying from 45.18 to 76.71% and 8.23 to 28.73% respectively. This variation depended on the studied group and the adopted scenario. This finding reiterates the fact that dietary exposure to a contaminant is the result of the contamination of food items by their consumption rates (FAO/WHO, 2009). Therefore, the higher the contamination level of the food item, the higher the contribution of that food item to the total contaminant intake, assuming similar food consumption rates. Similarly, if two food items had comparable contamination levels, the consumption of the food item will be the main determinant of the contribution of that item to the overall contaminant’s intake. This fact was demonstrated by the results of this study. Indeed, despite HM was reported to be consumed at variable rates by the different study groups, it was found to be the main contributor to MeHg intakes for all of them. Besides, CH and canned TU had comparable contamination levels (0.091 vs 0.103 µg/g). However, their contributions to total MeHg intake were different depending on their consumption rates. For example in the Qatari cohort, CH was more consumed than canned TU (200.8 ± 204 vs 94.67 ± 161), whereas, in the non-Qatari cohort, canned TU was more consumed than CH ( 178.1 ± 186 vs 95.5 ± 178). Thus, CH contributed 24.50% to the total MeHg intake in the Qatari cohort and 8.93% in the non-Qatari cohort, whereas canned TU contributed 8.82% to the total MeHg in the Qatari cohort and 30.11% in the non-Qatari cohort. This finding highlights the need to take into consideration both the contamination levels and the consumption rates when drawing regulatory guidelines and dietary advice (Soubra et al., 2009).

**Table 8:** The contribution of each fish species to the MeHg exposure for “all participants,” “Qatari” and “non-Qatari,” and “females of childbearing age” subgroups based on scenario 1

**Fish species**	**All participants** **(***n*** = 600)**		**Qatari cohort** **(***n*** = 336)**		**Non-Qatari cohort** **(***n*** = 264)**		**Female of childbearing subgroup** **(***n*** = 226)**		**High-protein diet group** **(***n*** = 118)**	
	**MeHg exposure**^***a***^	**%**^***b***^	***MeHg exposure***^***a***^	**%**^***b***^	**MeHg exposure**^***a***^	***%***^***b***^	**MeHg exposure**^***a***^	**%**^***b***^	**MeHg exposure**^***a***^	**%**^***b***^
**HM**	0.45	46.39	0.49	48.01	0.39	41.94	0.77	75.49	0.72	65.45
**SF**	0.003	0.30	0.004	0.39	0.002	0.22	0.00099	0.10	0.0009	0.08
**CH**	0.22	22.68	0.25	24.50	0.08	8.60	0.01	0.98	0.01	0.91
**SH**	0.11	11.3	0.11	10.78	0.09	9.68	0.005	0.49	0.03	2.73
**TU**	0.03	3.09	0.03	2.94	0.03	3.23	0.03	2.94	0.01	0.91
**TU (canned)**	0.11	11.3	0.09	8.82	0.28	30.11	0.18	17.65	0.31	28.18
**SM**	0.02	2.06	0.02	1.96	0.03	3.23	0.007	0.69	0.008	0.73
**SM (canned)**	0.004	0.41	0.005	0.49	0.004	0.43	0.01	0.98	0.001	0.09
**SB**	0.03	3.09	0.03	2.94	0.03	3.23	0.009	0.88	0.02	1.82
**Total**	0.97	100	1.02	100	0.93	100	1.02	100	1.10	100

^a^MeHg Exposure (µg/kg b.w./week): exposure to MeHg based on the consumption rates and contamination data of the fish species based on the average exposure

^b^%: percentage of contribution of the fish species to the total exposure to MeHg for the average exposure

**Table 9:** The contribution of each fish species to the MeHg exposure for “all participants,” “Qatari” and “non-Qatari,” and “females of childbearing age” subgroups based on scenario 2

**Fish species**	**All participants** **(*n* = 600)**		**Qatari cohort** **(*n* = 336)**		**Non-Qatari cohort** **(*n* = 264)**		**Female of childbearing subgroup** **(*n* = 226)**		**High-protein diet group** **(*n* = 118)**	
	**MeHg exposure**^***a***^	**%**^***b***^	**MeHg exposure**^***a***^	**%**^***b***^	**MeHg exposure**^***a***^	**%**^***b***^	**MeHg exposure**^***a***^	**%**^***b***^	**MeHg exposure**^***a***^	**%**^***b***^
**HM**	0.438	47.08	0.477	48.65	0.38	43.27	0.74921	76.71	0.701	66.53
**SF**	0.003	0.30	0.004	0.39	0.009	0.22	0.0009	0.10	0.001	0.08
**CH**	0.215	23.09	0.244	24.90	0.08	8.90	0.0097	1.00	0.010	0.93
**SH**	0.104	11.13	0.104	10.56	0.08	9.66	0.0047	0.48	0.028	2.68
**TU**	0.028	3.04	0.028	2.88	0.03	3.22	0.0282	2.89	0.009	0.89
**TU (canned)**	0.100	10.70	0.081	8.31	0.22	28.89	0.1629	16.68	0.281	26.64
**SM**	0.016	1.73	0.016	1.64	0.02	2.75	0.0056	0.58	0.006	0.61
**SM (canned)**	0.003	0.35	0.004	0.41	0.004	0.37	0.0080	0.82	0.001	0.08
**SB**	0.024	2.62	0.024	2.48	0.02	2.77	0.0072	0.75	0.016	1.54
**Total**	0.93	100	0.98	100	0.88	100	0.98	100	1.05	100

^a^MeHg Exposure (µg/kg b.w./week): exposure to MeHg based on the consumption rates and contamination data of the fish species based on the average exposure estimates

^b^%: percentage of contribution of the fish species to the total exposure to MeHg for the average exposure

The average, p75, and p95 of MeHg intake estimates based on the T-Hg contamination levels of the analyzed fish samples and using the two scenarios are presented in Tables 10 and 11. The average MeHg weekly intake estimates ranged between 0.75 (non-Qatari male group) to 1.15 (Qatari high-protein diet group) μg·kg^−1^·w^−1^, depending on the study group and the applied scenario. In addition, the intake estimates at the 75th and 95th percentiles ranged between 0.88 (non-Qatari male group) to 2.11 (Qatari high-protein diet group) μg·kg^−1^·w^−1^ and 2.01 (Qatari male group) to 3.65 (Qatari high protein diet group) μg·kg^−1^·w^−1^ respectively, depending also on the study group and the applied scenario. Besides, when applying EU permissible levels for estimating intakes, the results were alarming. The average, p75, and p95 intake estimates for all the study population groups significantly exceeded the TWI set by EFSA (1.3 μg·kg^−1^·w^−1^, *p* ≤ 0.05) (data not shown). This finding highlights that the regulatory limits set in Europe are not protective for the study sample.

**Table 10:** Estimated dietary exposure to MeHg expressed in µg/kg b.w./week for Qatari residents from fish consumption based on scenario 1

**Groups**	**Exposure (µg/kg b.w**^**a**^**/week) scenario 1**^**b**^								
	**All participants** **(*n* = 600)**			**Qatari cohort** **(*n* = 336)**			**Non-Qatari cohort** **(*n* = 264)**		
	**Average**	**P75**	**p95**	**Average**	**p75**	**p95**	**Average**	**p75**	**p95**
**All participants group** **(*n* = 600)**	0.97	1.20	2.81	1.02	1.26	3.06	0.93	1.05	2.50
**High-protein diet group** **(*n* = 118)**	1.10	2.01	3.45	1.15	2.11	3.65	1.05	1.90	3.25
**Male group** **(*n* = 174)**	0.83	0.94	2.12	0.88	0.98	2.09	0.79	0.93	2.36
**Female group** **(*n* = 426)**	1.01	1.27	3.03	1.08	1.32	3.24	0.95	1.14	2.81
**Female non-childbearing age subgroup** **(*n* = 200)**	1	0.91	2.97	0.80	1.33	3.41	0.90	1.07	2.33
**Female childbearing age subgroup** **(*n* = 226)**	1.02	1.25	3.19	1.04	1.31	3.47	0.98	1.15	2.97

^a^b.w. = body weight

^b^Scenario 1: 100% of T-Hg were assumed to be MeHg

**Table 11:** Estimated dietary exposure to MeHg expressed in µg/kg b.w./week for Qatari residents from fish consumption based on scenario 2

**Groups**	**Exposure (µg/kg b.w**^**a**^**/week)** **Scenario 2**^**b**^								
	**All participants** **(*n* = 600)**			**Qatari cohort** **(*n* = 336)**			**Non-Qatari cohort** **(*n* = 264)**		
	**Average**	**P75**	**p95**	**Average**	**p75**	**p95**	**Average**	**p75**	**p95**
**All participants group** **(*n* = 600)**	0.93	1.14	2.70	0.98	1.21	2.96	0.88	0.99	2.40
**High-protein diet group** **(*n* = 118)**	1.05	1.95	3.25	1.15	2.01	3.31	0.95	1.85	3.19
**Male group** **(*n* = 174)**	0.79	0.89	2.03	0.84	0.93	2.01	0.75	0.88	2.25
**Female group** **(*n* = 426)**	0.98	1.21	2.92	1.03	1.27	3.08	0.90	1.09	2.70
**Female non-childbearing age subgroup** **(*n* = 200)**	0.95	1.19	2.83	1.02	1.25	3.07	0.86	1.02	2.23
**Female childbearing age subgroup** **(*n* = 226)**	0.98	1.20	3.05	1.03	1.29	3.27	0.93	1.09	2.70

^a^b.w. = body weight

^b^Scenario 2: T-Hg were assumed to be MeHg using percentages reported in the literature

These results show that the average intake estimates were below the TWI set by EFSA (1.3 μg·kg^−1^·w^−1^) for all groups. Moreover, the p75 intake estimates were close to (scenario 2) or exceeded (scenario 1) the EFSA TWI for the female group and subgroups, and exceeded the TWI (in both scenarios) for participants on a high-protein diet. In addition, the p95 of the intake estimates exceeded the TWI for all groups in both scenarios. These findings could be explained by the fact that, at the average intake estimates, participants might have either consumed fish that had low contamination levels or consumed fish that have high contamination levels in moderation, whereas, at the highest percentiles, participants consumed more fish with higher contamination levels and/or had high consumption of fish with low levels of MeHg. Moreover, MeHg intake estimates from fish consumption were variably reported in the literature. The average MeHg intakes were comparable to those reported by Barone et al. (202), Domínguez-Morueco et al. (2022); higher than those reported by Ahmad et al. (2022), Sarvan et al. (2021), Vasconcellos et al. (2021), Petrova et al. (2020), Wei et al. (2018), Laird et al. (2017), Ahmad et al. (2016), EFSA (2012), Maycock and Benford (2007); and lower than those reported by Fasha et al. (2018). Although comparisons with data reported by other countries may provide useful benchmarking, such comparisons would be interpreted with caution, as results of dietary intake estimates may be different based on a certain number of factors (Kroes et al., 2002; Soubra et al., 2009). These include, but are not limited to, the method and the model used to evaluate dietary exposure, the limits of detection/quantification of the analytical technique, the approaches used in exposure assessment, and the degree of preparation of the foods included in the assessment, etc. (Soubra, 2008, Soubra et al., 2009). In addition, such as in this study, intake estimates help identify the specific foods that are mostly contributing to the dietary exposure to a contaminant (Kroes et al., 2002). This information is warranted since the contribution of a specific food item to contaminant exposure depends not only on the concentration of the contaminant in that particular food but also on the amount of that specific food typically consumed by the population (Kroes et al., 2002; Soubra et al., 2009; Soubra, 2008). Hence, food items that represent the major source of exposure to a contaminant in one country may not be the same in another one where the population has a different dietary pattern. Consequently, regulatory guidelines set in a country may not necessarily work for another country. This fact was confirmed by the results of this study where it was observed that when applying EU permissible levels MeHg intakes exceeded the TWI for all study groups. Therefore, identifying the foods that are contributing most to the contaminants remains essential to setting targeted regulatory guidelines.

### Risk characterization

Tables 12 and 13 present the hazard quotients (percentage to TWI) and the percentages of participants exceeding the TWI for the study groups. Results of this study showed that the HQs at the average and P75 intake estimates are lower than one for all the study groups, except for the “participants on high-protein diet” groups at both the average and p75 estimates (both scenarios) and “females” groups of the Qatari cohort at the p75 estimates (first scenario). Moreover, the HQs at the p95 estimates were above one for all groups. Besides, a substantial percentage of the participants had intakes exceeding the TWI, with the highest percentages reported in “participants on high-protein diet” groups, followed by “females of childbearing age” subgroups. Alarmingly, 3.2% of the females of childbearing age were pregnant and 3% were breastfeeding. These findings suggest that MeHg would pose a risk to the health of some fish consumers. Sensitivity to the toxic effects of MeHg is related to the age at which exposure occurs, and therefore, the risk would be different depending on the age of the concerned groups (ATSDR, 2022; EFSA, 2012; FAO/WHO, 2006). For instance, the developing fetus, infants, and children are considered to be the most vulnerable population subgroups because of the immature nervous system and rapid brain development (Fernandes Azevedo et al., 2012). Exposure to MeHg during pregnancy may affect the developing brain of the fetus yielding various toxic effects. The toxicity of MeHg to the developing brain was first described in Minamata, Japan, where consumption of fish with high concentrations of methyl mercury by pregnant women, which is not the case in this study, resulted in cerebral palsy in children; exposed women were affected minimally if at all (Harada, 1968). The vulnerability of the developing brain to MeHG is related to its lipophilicity, which enables it to cross the placenta and concentrate in the central nervous system (Fernandes Azevedo et al., 2012). Besides, the blood–brain barrier is not fully developed until the first year of age, which facilitates the movement of contaminants across this barrier (National Research Council (US), 2000). MeHg can adversely affect memory, attention, language, visual-spatial perception, and gross motor skills, and cause intelligence loss in children (Thurston et al., 2022). The resulting loss of intelligence from MeHg exposure would have long-term consequences as it would be associated with a decrease in productivity that persists over the entire lifetime (Trasande et al., 2005). This loss of productivity would pose a significant economic burden on the country in the long run. However, the effects on IQ are still controversial. A study showed that maternal prenatal blood mercury was not adversely associated with offspring IQ at 8 years provided the mother eats fish (Golding et al., 2017). Moreover, methyl mercury exposure was also associated with decreased sympathetic- and parasympathetic-mediated modulation of heart rate variability (Fernandes Azevedo et al., 2012). Moreover, MeHg can cause neurodegenerative diseases in adults. It is believed that the mechanisms are related to the increase in reactive oxygen species (ROS) (National Research Council (US), 2000). Oxidative stress has been associated with the etiology of neurodegenerative diseases such as Parkinson’s and Alzheimer’s diseases, but these mechanisms have yet to be fully recognized (Pardillo-Díaz et al., 2022). Besides, exposure to low doses of MeHg appears to affect the immune and reproductive systems (ATSDR, 2022). Although there are no data on the effects of MeHg on the immune and reproductive systems in humans, animal studies have demonstrated that MeHg had adverse effects on the immune-cell ratios, cellular responses, the developing immune system, reduced fertility, reduced size of infants in one birth, the reduced survival rate of fetuses, and fetus abnormalities (ATSDR, 2022). Moreover, exposure to very low doses of MeHg has been associated with increased blood pressure, acute myocardial infarction, coronary dysfunction, and atherosclerosis in humans (Roman et al., 2011). It is believed that the mechanisms are related to the increase in the reactive oxygen species (ROS), dysregulation of glutathione and catalase anti-oxidant effects, lipid peroxidation, platelet aggregation, and arteries sclerosis (Fernandes Azevedo et al., 2012; Grotto et al., 2010). The toxic effects of cardiovascular effects should not be overlooked since cardiovascular diseases are the leading cause of mortality and morbidity in Qatar (Al-Absi et al., 2021). An increment, yet small, in the prevalence of cardiovascular diseases would yield an increase in the economic burden of the disease, which would pose additional pressure on the healthcare system. On the other hand, the impact of fish consumption on cardiovascular disease (CVD) is controversial. A metanalysis conducted by Zhang et al. showed that higher fish consumption was significantly associated with a lower CVD incidence and CVD mortality rates (Zhang et al., 2020). A recent cohort study showed that fish intake of at least 175 g (2 servings) weekly is associated with lower risk of major CVD and mortality among patients with prior CVD, but not in the general population (Mohan et al., 2021). Finally, despite the negative effects of MeHg consumption, fish consumption, in general, seems to have overweighing positive results on health and development, due to nutritional benefits. Therefore, a benefit/risk ratio should be considered when developing dietary advice.

**Table 12:** Risk characterization of MeHg for the studied population groups based on scenario1

**Groups**	All participants				Qatari cohort				Non-Qatari cohort			
	***HQ***^***a***^				***HQ***^***a***^				***HQ***^***a***^			
	**Average**	**P75**	**P95**	**%**^**b**^	**Average**	**p75**	**p95**	**%**^**b**^	**Average**	**p75**	**p95**	**%**^**b**^
**All participants** **(*n* = 600)**	0.74	0.92	2.16	19.67	0.78	0.96	2.35	23.21	0.69	0.80	1.92	18.94
**High-protein diet group** **(*n* = 118)**	0.84	1.54	2.65	25.42%	0.88	1.62	2.81	12.71	0.81	1.46	2.5	12.71
**Male group** **(*n* = 174)**	0.63	0.72	1.63	4.6	0.68	0.75	1.61	2.3	0.61	0.72	1.82	2.3
**Female group** **(*n* = 426)**	0.78	0.98	2.33	18.78	0.83	1.02	2.49	10.56	0.73	0.88	2.16	8.21
**Female non-childbearing age subgroup** **(*n* = 200)**	0.77	0.70	2.28	15	0.62	1.02	2.62	8.5	0.69	0.82	1.79	6.5
**Female childbearing age subgroup** **(*n* = 226)**	0.8	0.96	2.45	22.12	0.69	1.01	2.67	13.27	0.75	0.88	2.28	8.8

^a^*HQ* hazard quotient obtained by dividing the EWI over the TWI set by EFSA (1.3 µg/kg b.w. d/ week)

^b^The percentage represents the ratio between the number of consumers exceeding the EWI Value and the total number of subjects

**Table 13:** Risk characterization of MeHg for the studied population groups based on scenario 2

**Groups**	All participants				Qatari cohort				Non-Qatari cohort			
	***HQ***^***a***^				***HQ***^***a***^				***HQ***^***a***^			
	**Average**	**P75**	**P95**	**%**^**b**^	**Average**	**p75**	**p95**	**%**^**b**^	**Average**	**p75**	**p95**	**%**^**b**^
All participants (*n* = 600)	0.72	0.88	2.08	18.33	0.75	0.93	2.28	23.21	0.68	0.76	1.85	18.94
High-protein diet (*n* = 118)	0.81	1.50	2.50	22.88%	0.88	1.55	2.55	12.71	0.73	1.42	2.45	10.17
Male group (*n* = 174)	0.61	0.68	1.56	4.6	0.65	0.72	1.55	2.3	0.58	0.68	1.73	2.3
Female group (*n* = 426)	0.75	0.93	2.25	17.61	0.79	0.98	2.37	9.86	0.69	0.84	2.08	7.75
Female non-childbearing age subgroup (*n* = 200)	0.73	0.91	2.18	14	0.78	0.96	2.36	8.5	0.66	0.78	1.72	5.5
Female childbearing age subgroup (*n* = 226)	0.75	0.92	2.35	20.8	0.79	0.99	2.52	11.94	0.72	0.84	2.08	8.8

^a^*HQ* hazard quotient obtained by dividing the EWI over the TWI set by EFSA (1.3 µg/kg b.w. d/week)

^b^The percentage represents the ratio between the number of consumers EWI and the total number of subjects

### Risk management

The threshold contamination level of fish based on the current consumption pattern of females of the childbearing age subgroup yielded a value of 0.12 µg/g, which is much lower than that set by the EU guidelines. This can be explained by the difference in the fish-eating patterns between Qatar and the European countries from one side, and to the fish species that are consumed from the other side. Besides, the limits defined in European legislation may also be related to contamination levels/occurrence data in fish marketed and fished in the European region, which can be different from those in the Qatar region. Furthermore, based on the current contamination levels, HM consumption should be restricted to around 200 g/week (Table 14) and/or canned TU to around 300 g/week to keep intakes within the TWI.

**Table 14:** Safe fish consumption rates per week (CONS) in g/week (portion) for “all participants,” “female of childbearing age” subgroup, and high-protein diet” group for the fish species that is/are the main contributor(s) to MeHg exposure

**Fish species**	**All participants**	**Female childbearing age subgroup**	**High-protein diet group**
	**Fish CONS (g/w)**	**Fish CONS (g/w)**	**Fish CONS (g/w)**
Scenario 1^a^			
** HM**	225.37	202.19	197.5
** TU (canned)**	-	-	343.09
Scenario 2^b^			
** HM**	231.65	207.82	203
** TU (canned)**	-	-	378.74

^a^Scenario 1: the fish species was assumed to contribute by 100% to the average MeHg exposure. The safe consumption rates/w were calculated based on the fish species that was found to be contributing most to the MeHg exposure and using the highest contamination data observed for that fish species

^b^Scenario 2: the fish species was assumed to contribute to the average MeHg exposure by the percentage observed in this study. The safe consumption rates/w were calculated based on the fish species that was found to be contributing most to the MeHg exposure and using the highest contamination data observed for that fish species

### Study limitations

This study has many strengths such as being the first study in Qatar that assessed the health risks of MeHg resulting from fish consumption based on fish consumption data collected from a dietary survey and fish contamination data collected from fish analysis, and using a representative sample size of the adult Qatari residents. In addition, the use of individual fish species composite samples approach for analyses has the advantages to be able to estimate the contribution of individual foods, in this case fish species, to exposures as well as the greater flexibility in calculating dietary exposures for various segments of the population, provided appropriate food consumption information is available (FAO/WHO, 2009). Moreover, the use of detailed fish consumption survey and the disaggregated approach when estimating the exposure to MeHg would have yielded a more realistic determination of MeHg exposure level (FAO/WHO, 1997). Finally, although it is acknowledged that a mean may be a poor indicator of the central trend of distribution, particularly when that distribution is markedly skewed (which is often the case for contamination data), its use in intake calculations provides a realistic and appropriate estimation of long-term exposure, because these intakes are compared with the reference toxicological intakes established over an entire lifetime (FAO/WHO, 1997).

This study has also some limitations. First, the risk estimates were based on the mercury concentration of raw samples and not of “as consumed” samples. This might have introduced some uncertainties in the estimates since controversial effects of cooking on mercury concentration in fish were reported in the literature, where some studies reported no effects, while others reported an increase or a decrease in the concentration depending on the fish species and cooking process (Schmidt et al., 2015; Domingo, 2010; Farias et al., 2010; Perreló et al., 2008; burger et al., 2003, Chicourel et al., 2001; Morgan et al., 1997). Besides, the fish samples were analyzed for their T-Hg content and not MeHg. However, T-Hg is the sum of all chemical forms of mercury, including MeHg. Analyzing fish tissues for total mercury is less expensive than analyzing the individual chemical forms of mercury, such as MeHg. Moreover, fish tissues are commonly analyzed for total mercury, and many international agencies have therefore developed standards based on total mercury levels. In addition, this approach is acceptable given that most of the mercury present in fish tissue is in the form of methylmercury (EFSA, 2012). Besides, in this study, two scenarios were adopted to account for this limitation and reduce uncertainties in the estimates. In the first scenario, which is the worst-case scenario, all T-Hg was considered to be MeHg without applying any conversion factor. In the second scenario, a conversion factor was used to convert the T-Hg to MeHg based on current literature. Moreover, the study has a cross-sectional design, and fish consumption and contamination data were collected at one point in time. This design does not take into consideration seasonal variations in both fish contamination and consumption pattern. However, the fish consumption survey was designed as a fish frequency questionnaire where participants had to report their usual fish eating pattern based on a year period of time (FAO/WHO, 2009). Therefore, reported fish consumption represents the usual consumption pattern of the participants over the seasons. Finally, the fish consumption survey was filled out online by the participants without any professional intervention or support. This might have led to an over/underestimation of the reported fish consumption.

Despite these limitations, this study provided baseline information on MeHg concentrations in the most commonly consumed fish species in Qatar. It also yielded important information on the risks that MeHg poses to the health of fish consumers and shed the light on the need to consider fish consumption patterns when drafting national standards and guidelines.

## Conclusion

The present study aimed at determining the health risks of MeHg for adult Qatari residents from fish consumption based on actual fish consumption and contamination data of the most consumed fish species. Results of this study revealed that, based on the collected fish consumption surveys, fish is the main source of protein in Qatar for both Qatari and non-Qatari residents. Moreover, the results of this study showed that all of the analyzed fish species collected from the Qatari market contained T-Hg, and subsequently MeHg, within permissible levels set by European Union regulations. Based on the analytical determination of T-Hg in the most consumed fish species and the food consumption data, the current MeHg TWI is likely to be exceeded by a proportion of fish consumers, including females of childbearing age. This finding highlighted the need for setting national threshold values based on fish consumption patterns, elaborating advice about eating fish for those who are following a high-protein diet as well as for those who might become or are pregnant or breastfeeding and children ages 1–11 years based on risk/benefit ratio, and routine monitoring of fish. It also shed the light on the need to conduct bio-monitoring studies to take into account the various sources of exposure and have a more comprehensive picture of the exposure to MeHg.

## Data Availability

The datasets generated during and/or analyzed during the current study are not publicly available due to authorship reasons but are available from the corresponding author on reasonable request. All authors have read, understood, and have complied as applicable with the statement on “Ethical responsibilities of Authors” as found in the Instructions for Authors and are aware that with minor exceptions, no changes can be made to authorship once the paper is submitted.
